# Design, synthesis and biological potentials of novel tetrahydroimidazo[1,2-a]pyrimidine derivatives

**DOI:** 10.1186/s13065-017-0245-9

**Published:** 2017-02-09

**Authors:** Jyoti Rani, Monika Saini, Sanjiv Kumar, Prabhakar Kumar Verma

**Affiliations:** Department of Pharmaceutical Sciences, Maharshi Dayanand University, Rohtak, Haryana 124001 India

**Keywords:** Pyrimidine derivatives, Antimicrobial, Antioxidant and anticancer activity

## Abstract

**Background:**

A novel series of 5-(substituted aldehyde)-7-methyl-3-oxo-*N*-phenyl-2-((3,4,5,6-tetrahydroxytetrahydro-2*H*-pyran-2-yl)methylene)-1,2,3,5-tetrahydroimidazo[1,2-*a*]pyrimidine-6-carboxamide analogues (**1**–**24**) was synthesized using the Biginelli condensation.

**Results and discussion:**

The synthesized compounds were screened for their in vitro antimicrobial potential against Gram (positive and negative) bacterial and fungal strains by tube dilution technique. In the series, compound **15** exhibited significant antimicrobial activity against *Candida albicans* and *Aspergillus niger* with MIC value = 1.04 × 10^−2^ µM/ml and compound **2** was found to be most active antioxidant agent with IC_50_ value = 46.31 using DPPH assay. Anticancer activity results indicated that compound **23** displayed better anticancer activity against human breast cancer cell line (MCF-7) with GI_50_ value = 34.78 using SRB assay.

**Conclusions:**

All synthesized derivatives exhibited good antimicrobial, antioxidant and anticancer activity using specific method and compared with standard drugs, especially compounds **2, 15** and **23** displayed more activity than reference drugs. Structure activity relationship demonstrated that presence of electron releasing groups of the synthesized compounds enhanced the antibacterial activity against *Escherichia coli* as well as antioxidant activity and electron withdrawing groups improved the antimicrobial as well as anticancer activity against human breast (MCF-7) cancer cell line.

## Background

Pyrimidines are obtained from the various natural resources and synthethic reaction in medicinal chemistry [1]. They are also known as *m*-diazine or 1,3-diazone can be considered as cyclic amine. Heterocyclic compounds are used in agricultural and medicinal reasons using biological and chemical studies. Pyrimidine derivatives play a vital role in several biological activities i.e. antihypertensive, anticancer, antimicrobial, anti-inflammatory, antifungal, analgesic, antioxidant, anticonvulsant and antiviral [2]. Antimicrobials agents are one of the most important weapons in the resistance of infection caused by bacterial strains [3]. In the past few years, increase the resistance of microorganisms toward antimicrobial agents become a serious health problem so there is a need of safe, potent and novel antimicrobial agents [4]. Pyrimidine derivatives showed most antimicrobial activity against Gram +ve and Gram –ve microorganism [5]. At that time, many antimicrobial drugs are present in the market but due to the indiscriminate use of antimicrobial agents often followed the development of resistant strains of microorganism so there is a need for the development of new class of active antimicrobial drugs with lesser or no side effects [6]. Pyrimidine agents recently attracted medicinal chemist in exploring their potential as antioxidant agents. Oxidative stress appears to play an important role in many human diseases, including cancers. The use of antioxidants in pharmacology is intensively studied, particularly for stroke and neurodegenerative diseases [7]. Antioxidants are the agents that neutralize free radicals, which scavenge reactive oxygen species may be high potent value in preventing the onset and propagation of oxidative diseases like neurovascular, autoimmune and cardiovascular diseases [8].

Cancer is one of the most serious medical problem and second leading cause of death in the world, characterized by a deregulation of the cell cycle which mainly results in a progressive loss of cellular differentiation and uncontrolled cellular growth. The current situation highlights the need for discovery and development of small molecule anticancer drugs with improved tumor selectivity, efficacy and safety remains desirable [9]. Many pyrimidine derivatives were reported to be active against various forms of cancer. Due to less effective, more side effect and lack of a broad range of anticancer agents there is a need of anticancer agents have motivated the idea of researchers toward the discovery of novel anticancer agents [10]. Owing to the pharmacological significance of pyrimidine derivatives so, we have planned to synthesize some new pyrimidine derivatives and evaluate for their antimicrobial, antioxidant and anticancer activities.

## Results and discussion

### Chemistry

**Scheme 1:**
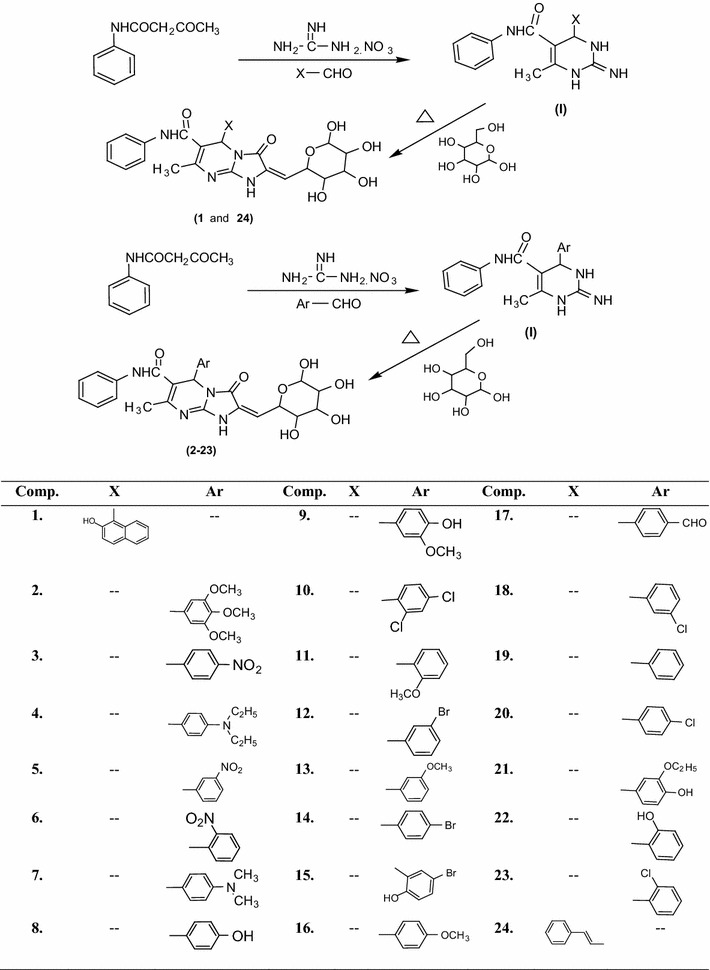
Synthesis of 5-(substituted aldehyde)-7-methyl-3-oxo-*N*-phenyl-2-((3,4,5,6-tetrahydroxytetrahydro-2*H*-pyran-2-yl)methylene)-1,2,3,5-tetrahydroimidazo[1,2-*a*]pyrimidine-6-carboxamide analogues

In the research work, we have synthesized new series of 5-(substituted aldehyde)-7-methyl-3-oxo-*N*-phenyl-2-((3,4,5,6-tetrahydroxytetrahydro-2*H*-pyran-2-yl)methylene)-1,2,3,5-tetrahydroimidazo[1,2-*a*]pyrimidine-6-carboxamide analogues using the Biginelli condensation and synthetic steps of this series showing in Scheme 1. The physiochemical properties (molecular formula; molecular weight; melting points; percentage yield etc.) of the synthesized analogues are presented in Table 1. The chemical structures of the synthesized compounds were confirmed by ^1^H/^13^C-NMR, FT-IR, Mass spectral and elemental analysis studies. The elemental analysis results of synthesized compounds were within ±0.4% of the theoretical values.

**Table 1:** The physicochemical properties of the synthesized analogous

Comp.	M. Formula	M. Wt.	m.p. (°C)	R_*f*_ value^a^	% yield
1	C_30_H_28_N_4_O_8_	572	121–123	0.58	86
2	C_29_H_32_N_4_O_10_	596	169–171	0.31	83
3	C_26_H_25_N_5_O_9_	551	159–161	0.53	80
4	C_30_H_35_N_5_O_7_	577	150–153	0.68	84
5	C_26_H_25_N_5_O_9_	551	122–124	0.63	91
6	C_26_H_25_N_5_O_9_	551	159–161	0.51	64
7	C_28_H_31_N_5_O_7_	549	161–163	0.56	88
8	C_26_H_26_N_4_O_8_	522	170–172	0.61	84
9	C_27_H_28_N_4_O_9_	552	146–148	0.41	80
10	C_26_H_24_Cl_2_N_4_O_7_	574	148–150	0.42	83
11	C_27_H_28_N_4_O_8_	536	174–176	0.45	78
12	C_26_H_25_BrN_4_O_7_	584	144–146	0.66	72
13	C_27_H_28_N_4_O_8_	536	148–150	0.39	78
14	C_26_H_25_BrN_4_O_7_	584	155–157	0.38	72
15	C_26_H_25_BrN_4_O_8_	601	119–121	0.62	90
16	C_27_H_28_N_4_O_8_	536	149–151	0.47	91
17	C_27_H_26_N_4_O_8_	534	140–142	0.25	79
18	C_26_H_25_ClN_4_O_7_	540	150–153	0.59	73
19	C_26_H_26_N_4_O_7_	506	144–146	0.47	83
20	C_26_H_25_ClN_4_O_7_	540	151–153	0.55	75
21	C_28_H_30_N_4_O_9_	566	146–148	0.66	85
22	C_26_H_26_N_4_O_8_	522	100–102	0.61	74
23	C_26_H_25_ClN_4_O_7_	540	141–143	0.56	77
24	C_28_H_28_N_4_O_7_	532	143–145	0.53	81

^**a**^TLC mobile phase-Benzene

#### Antimicrobial activity

The in vitro antimicrobial activity of synthesized compounds against Gram-positive bacteria: *Staphylococcus aureus* (MTCC 3160), *Bacillus subtilis* (MTCC 441), Gram-negative bacterium: *Escherichia coli* (MTCC 443) and fungal: *Candida albicans* (MTCC 227) and *Aspergillus niger* (MTCC 281) strains was examined by tube dilution method [11]. Norfloxacin and fluconazole used as standard for antibacterial and antifungal activities respectively. Dilutions of test and standard compounds were prepared in double strength nutrient broth for bacterial strains and sabouraud dextrose broth for fungal strains [12]. The samples were incubated at 37 ± 1 °C for 24 h (for bacterial species), at 25 ± 1 °C for 7 days (*A. niger*) and at 37 ± 1 °C for 48 h (*C. albicans*) respectively and the results were recorded in terms of MIC (the lowest concentration of test substance which inhibited the growth of microorganisms). In case of Gram positive bacteria, compounds **12** and **14** (MIC_sa_ = 2.14 × 10^−2^ µM/ml) having significant activity against *S. aureus* and compound **18** (MIC_bs_ = 0.58 × 10^−2^ µM/ml) exhibited most potent against *B. subtilis*. In case of Gram negative bacterium, compound **21** (MIC_ec_ = 1.10 × 10^−2^ µM/ml) displayed more potent activity against *E. coli.* Compound **15** (MIC_ca & an_ = 1.04 × 10^−2^ µM/ml) was found to be most potent against *C. albicans* and *A. niger*. These compounds may be taken as lead to discovery novel antimicrobial agents. The presented results are showing in Table 2.

**Table 2:** Antimicrobial activity (MIC = µM/ml) of the synthesized analogous

Comp.	Minimum inhibitory concentration (MIC)				
	Bacterial strains			Fungal strains	
	*S. aureus*	*B. subtilis*	*E. coli*	*C. albicans*	*A. niger*
1	2.19	2.19	2.19	2.19	1.09
2	4.19	2.10	2.10	1.05	1.05
3	2.27	2.27	2.27	1.13	1.13
4	4.33	4.33	8.67	2.17	1.08
5	9.07	1.13	9.07	2.27	1.13
6	2.27	2.27	2.27	2.27	1.13
7	2.28	1.14	2.28	2.28	1.14
8	2.39	2.39	2.39	2.39	1.20
9	2.26	2.26	2.26	2.26	1.13
10	2.18	2.18	2.18	2.18	1.09
11	2.33	1.17	2.33	2.33	1.17
12	2.14	2.14	2.14	2.14	2.14
13	9.33	2.33	9.33	2.33	2.33
14	2.14	2.14	2.14	2.14	2.14
15	4.16	1.04	2.08	1.04	1.04
16	4.66	1.17	2.33	2.33	2.33
17	2.34	1.17	2.34	2.34	1.17
18	4.63	0.58	2.31	2.31	2.31
19	2.47	1.24	2.47	2.47	1.24
20	2.31	1.16	2.31	1.16	2.31
21	2.21	1.10	1.10	1.10	1.10
22	2.39	2.39	2.39	1.20	1.20
23	4.63	1.16	2.31	2.31	2.31
24	2.35	1.17	2.35	1.17	1.17
Std.	0.47^a^	0.47^a^	0.47^a^	0.50^b^	0.50^b^

^a^Norfloxacin

^b^Fluconazole

#### Antioxidant activity

The antioxidant activity of the synthesized compounds was evaluated with spectrophotometrically using free radical scavenging DPPH assay. The DPPH is a stable free radical with maximal absorption at 517 nm and is reduced to a corresponding hydrazine when it reacts with hydrogen donors. When DPPH reacts with an antioxidant agent, it can donate hydrogen get reduced and deep violet colour of DPPH change to yellow, showing a considerable decreased in absorption at 517 nm. DPPH solution (3 μg/ml) was prepared in methanol (methanol: DPPH in 1:1) for blank reference. Four types of dilutions were prepared in the methanol of the synthesized derivatives and standard (ascorbic acid) in the concentration of 25, 50, 75 and 100 μg/ml and then 1 ml of each concentration was added to 1 ml of DPPH solution. The solution mixture was shaken vigorously and kept in dark place for 30 min at room temperature and absorbance was measured by UV at 517 nm [13]. Free radical DPPH inhibition in percentage (%) was calculated as follows:

1 $$\% \,{\text{Inhibiton}}\,{ = }\,\frac{{{\text{A}}_{\text{Blank}} - {\text{A}}_{\text{Sample}} }}{{{\text{A}}_{\text{Blank}} }}\, \times \,100 ,$$

where, A_Blank_ = absorbance of the blank reaction, A_Sample_ = absorbance of the test compound

**Fig. 1:**
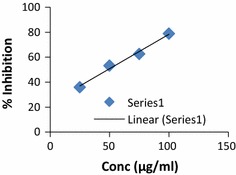
Standard graph of ascorbic acid

**Fig. 2:**
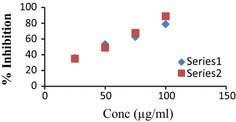
Graph of potent antioxidant compounds **2** and **16**

**Fig. 3:**
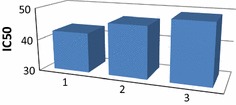
IC_50_ values of compounds **2** and **16** compared to ascorbic acid

IC_50_ value was calculated from the graph plotted between % inhibition and synthesized compound (Figs. 1, 2, 3). Antioxidant activity demonstrated, compounds **2** and **16** exhibited excellent activity at absorbance 517 nm with IC_50_ values = 46.31 and 48.81 respectively and compared with ascorbic acid as standard drug. These compounds may be used as a lead for development of new antioxidant agents. The presented results are showing in Table 3.

**Table 3:** Antioxidant activity of the synthesized analogous

Comp.	% Inhibition				IC_50_ µg/ml
	25 µg/ml	50 µg/ml	75 µg/ml	100 µg/ml	
1	30.56	42.68	55.52	76.45	60.30
2	37.25	51.23	67.34	89.45	46.31
3	20.62	35.93	56.24	69.85	68.90
4	15.71	33.96	43.59	65.21	78.60
5	21.73	37.39	58.72	73.24	65.60
6	25.65	30.95	51.34	67.28	72.70
7	14.59	24.78	47.64	59.45	83.20
8	26.34	37.31	55.28	72.52	65.80
9	32.62	48.28	65.21	82.16	51.83
10	28.89	45.85	60.27	72.56	59.20
11	26.73	47.19	63.81	79.34	56.40
12	17.62	42.95	56.57	68.28	67.80
13	32.47	47.61	64.92	78.52	53.06
14	19.53	41.63	61.57	74.82	63.30
15	22.68	39.91	57.74	71.73	65.40
16	35.95	53.23	62.58	78.84	48.81
17	24.64	43.98	61.37	74.81	60.70
18	32.94	48.92	59.38	72.49	55.90
19	19.62	43.81	61.52	74.49	62.60
20	21.71	34.61	56.70	75.82	66.24
21	15.25	33.41	47.43	65.51	77.00
22	19.26	33.16	50.16	69.25	72.90
23	23.67	47.28	65.11	78.26	57.65
24	23.68	47.28	56.14	72.61	62.60
Ascorbic acid	39.52	55.74	68.25	93.61	42.52

#### Anticancer activity

**Fig. 4:**
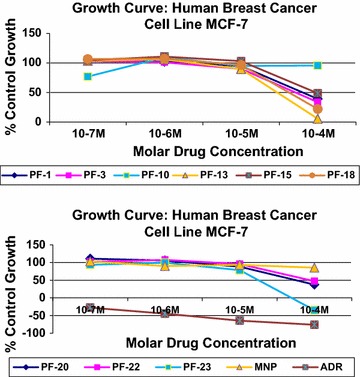
Graph plotted between tested compound and standard drug

In vitro anticancer potential of the newly synthesized 5-(substituted aldehyde)-7-methyl-3-oxo-*N*-phenyl-2-((3,4,5,6-tetrahydroxytetrahydro-2*H*-pyran-2-yl)methylene)-1,2,3,5 tetrahydroimidazo[1,2-*a*]pyrimidine-6-carboxamide analogues were carried out by sulforhodamine B (SRB) assay against human breast (MCF-7) cancer cell line. All synthesized compounds submitted to screen have been tested initially at dose (10^−7^–10^−4^ M) at anticancer drug screening facility (ACDSF) at ACTREC, Tata Memorial Centre, and Mumbai. Among them, compound **23** was found to be most potent anticancer agent at dose 10^−4^ M against human breast (MCF-7) cancer cell line and comparable with adriamycin as standard (Tables 4, 5). Graph plotted between tested compound and standard drug presented in Fig. 4.

**Table 4:** Percentage (%) control growth against human breast cancer cell line MCF-7

	Human breast cancer cell line MCF-7															
	% Control growth															
	Molar drug concentrations															
	Experiment 1				Experiment 2				Experiment 3				Average values			
	10^−7^M	10^−6^M	10^−5^M	10^−4^M	10^−7^M	10^−6^M	10^−5^M	10^−4^M	10^−7^M	10^−6^M	10^−5^M	10^−4^M	10^−7^M	10^−6^M	10^−5^M	10^−4^M
1	106.9	101.9	100.7	38.0	106.9	97.2	90.7	38.6	102.8	106.8	92.6	41.0	105.6	102.0	94.7	39.2
3	111.3	91.2	83.4	29.3	107.9	101.8	90.2	33.2	90.1	110.2	96.3	38.6	103.1	101.1	90.0	33.7
10	111.2	106.9	87.6	80.0	45.3	115.4	88.8	93.4	74.4	106.6	108.4	113.4	77.0	109.6	94.9	95.6
13	101.0	104.4	84.4	−1.9	107.3	107.7	92.6	1.9	102.5	105.9	91.9	16.8	103.6	106.0	89.6	5.6
15	96.6	100.8	99.0	41.7	106.8	114.1	102.6	49.0	110.1	117.3	107.4	55.3	104.5	110.8	103.0	48.7
18	94.69	99.7	92.55	22.66	117.9	108.6	92.21	22.27	107	113.6	105	21.09	106.5	107.3	96.59	22.01
20	110.7	96.6	78.2	34.8	114.2	112.1	87.7	35.3	107.7	108.6	99.0	42.1	110.9	105.8	88.3	37.4
22	102.1	107.3	91.5	46.9	99.6	103.7	91.1	48.3	104.0	111.1	104.4	46.1	101.9	107.4	95.7	47.1
23	100.2	95.4	77.2	−*44.5*	104.0	101.5	76.8	−*43.1*	76.3	101.6	81.4	−*17.1*	93.5	99.5	78.5	−*34.9*
MNP	105.0	97.0	99.8	86.2	101.6	108.5	90.4	89.1	102.4	64.1	90.4	81.0	103.0	89.9	93.5	85.5
ADR	−34.5	−46.5	−63.5	−79.5	−62	−49.7	−67	−79.3	13.69	−38.2	−63.6	−69.9	−27.6	−44.8	−64.7	−76.2

The significance of italic values was found to be most active against human breast (MCF-7) cancer cell line due to the presence of electron withdrawing groups (*o*-Cl) on benzylidene portion

**Table 5:** Anticancer activity of the selected synthesized analogous

MCF-7	µMolar drug concentrations		
	LC_50_	TGI	GI_50_^a^
1	>100	>100	82.82
3	>100	>100	75.3
10	NE	NE	NE
13	>100	>100	54
15	>100	>100	97.9
18	>100	>100	66.8
20	>100	>100	80.08
22	>100	>100	94.5
23	>100	73.05	34.78
MNP	>100	>100	>100
ADR	18.01	<0.1	<0.1

Where ^a^GI_50_ ≤ µMolar is considered to be active, LC_50_, concentration of drug causing 50% cell kill; GI_50_, concentration of drug causing 50% inhibition of cell growth; TGI, concentration of drug causing total inhibition of cell growth; ADR adriamycin, positive control compound

#### SAR (structure activity relationship) studies

**Fig. 5:**
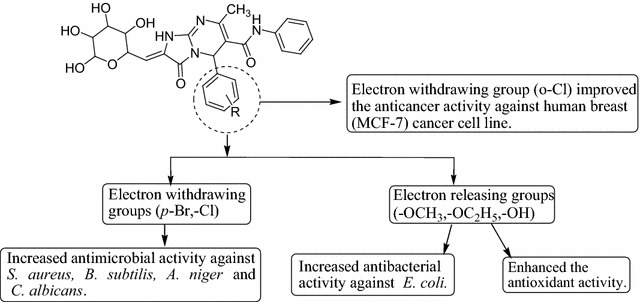
Structural requirements for the antimicrobial, anticancer and antioxidant activities of synthesized derivatives

From the antimicrobial, antioxidant and anticancer activities results of the synthesized 5-(substituted aldehyde)-7-methyl-3-oxo-*N*-phenyl-2-((3,4,5,6-tetrahydroxytetrahydro-2*H*-pyran-2-yl)methylene)-1,2,3,5-tetrahydroimidazo[1,2-*a*]pyrimidine-6-carboxamide analogues, the subsequent structure activity relationship can be derived in Fig. 5.

- Presence of electron releasing groups (–OC_2_H_5_, –OH, Compound **21**) on benzylidene portion improved the antibacterial activity of the synthesized compounds against *E. coli.*
- Presence of electron withdrawing groups (–Br, –Cl, Compounds **12, 14, 15** and **18**) on benzylidene portion improved the antimicrobial activity of the synthesized compounds against *S. aureus, B. subtilis, A. niger* and *C. albicans.*
- Presence of electron releasing groups (trimethoxy and *p*-OCH_3_, Compounds **2** and **16**) on benzylidene portion enhanced the antioxidant activity.
- Presence of electron withdrawing group (*o*-Cl, Compound **23**) on benzylidene portion improved the anticancer activity of the synthesized compounds against human breast (MCF-7) cancer cell line.

### Experimental section

Synthesized pyrimidine derivatives followed the general procedure discussed in synthetic (Scheme 1). All reagents and solvents used in study were of both laboratory and analytical grade and procured from commercial market. Reaction steps forward was observed by thin layer chromatography making use of commercial silica gel plates. Melting points were tested in open capillary tubes method. ^1^H nuclear magnetic resonance (^1^H-NMR) spectral study demonstrated by Bruker Avance 400 NMR spectrometer in appropriate DMSO-deuterated solvents and are expressed in parts per million (δ, ppm) downfield from tetramethyl silane (internal standard). ^1^H-NMR data are given as multiplicity (s, singlet; d, doublet; t, triplet; m, multiplet) and number of protons. Infrared (IR) spectra were recorded on Bruker 12060280, Software: OPUS 7.2.139.1294 spectrophotometer.

#### General procedure for synthesized pyrimidine analogues

##### Step 1: intermediate-I

A mixture of 3-oxo-*N*-phenylbutanamide (0.02 mol), guanidine nitrate (0.030 mol) and corresponding aldehyde (0.02 mol) in the round bottom flask with 100 ml methanol and then added aluminum chloride (0.006 mol) with 4–5 drops of concentrated hydrochloric acid after that the reaction mixture was refluxed for 10–11 h. before completion of the reaction we had been checked the reaction with every 30 min by TLC plats with suitable solvent system (benzene). After completion of the reaction the reaction mixture was cooled at room temperature and poured into ice cold water with vigorous stirring, filtered and recrystallized with methanol [11].

##### Step 2: final analogues (1–17)

The intermediate-1 (0.02 mol, *synthesized in previous step*-*1*), sodium benzoate (4 gm), 6-(hydroxymethyl)-tetrahydro-2*H*-pyran-2,3,4,5-tetraol (0.02 mol), ethyl acetoacetate (15 ml), glacial acetic acid (40 ml) and monochloroacetic acid (0.030 mol) were taken in round bottom flask and refluxed with for 6–7 h (controlled temperature at 140–142 °C) before completion of the reaction, we had been checked the reaction with every 30 min by TLC plats with suitable solvent system (benzene). After completion of the reaction the reaction mixture was cooled at room temperature and poured into ice cold water to yielded solid precipitate, filtered and recrystallized with methanol.

### Spectral analysis determined by

**FT-IR (KBr pellets, cm** ^**−1**^ **) and** ^**1**^ **H-NMR/** ^**13**^ **C-NMR (DMSO-d**_**6**_ **, δ ppm),** stretching = st.; pyrimidine nucleus = pn

**Compound 1** ***(5*** **-** ***(2*** **-** ***Hydroxynaphthalen*** **-** ***1*** **-** ***yl)*** **-** ***7*** **-** ***methyl*** **-** ***3*** **-** ***oxo*** **-** ***N*** **-** ***phenyl*** **-** ***2*** **-** ***((3,4,5,6*** **-** ***tetrahydroxytetrahydro*** **-** ***2H*** **-** ***pyran*** **-** ***2*** **-** ***yl)methylene)*** **-** ***1,2,3,5*** **-** ***tetrahydroimidazo[1,2*** **-** ***a]pyrimidine*** **-** ***6*** **-** ***carboxamide)*** **IR:** {3060 (C–H st.), 1596 (C=C st.), 712 (C–C st.) of aromatic ring}, 1630 (C=O st.,), 3340 (N–H st., 2° amide), {1630 (N=CH st.), 1313 (C–N st.) of pn}, 2831 (C–H st., cyclic ether), 1093 (C–O–C st., aryl ether), 3340 (O–H st., polyhydroxy); ^**1**^ **H-NMR (DMSO-d**_**6**_ **, δ ppm):** 7.16–7.65 (m, 11H, Ar–H), 2.13 (s, 1H, NH), 8.03 (s, 1H, NH of 2^o^ amide), 3.47-4.26 (m, 5H, CH of tetrahydropyran), 2.20 {s, 4H, (OH)_4_}. ^**13**^ **C-NMR (DMSO-d**_**6**_ **, δ ppm):** 24.5, 51.3, 77.4, 78.3, 98.7, 115.3, 118.4, 121.5, 130.6, 146.3, 163.4, 121.3, 123.4, 122.8, 137.4, 127.1, 128.8, 133.6, 153.6, 119.3; **MS ES** + (ToF): *m/z* 572 [M^+^+1].

**Compound 2** ***(7*** **-** ***Methyl*** **-** ***3*** **-** ***oxo*** **-** ***N*** **-** ***phenyl*** **-** ***2*** **-** ***((3,4,5,6*** **-** ***tetrahydroxytetrahydro*** **-** ***2H*** **-** ***pyran*** **-** ***2*** **-** ***yl)methylene)*** **-** ***5*** **-** ***(3,4,5*** **-** ***trimethoxyphenyl)*** **-** ***1,2,3,5*** **-** ***tetrahydroimidazo[1,2*** **-** ***a]pyrimidine*** **-** ***6*** **-** ***carboxamide)*** **IR:** {3062 (C–H st.), 1596 (C=C st.), 694 (C–C st.,) of aromatic ring}, 1630 (C = O st.), 3321 (N–H st., 2^o^ amide), {1630 (N=CH st.), 1244 (C–N st.) of pn}, 2779 (C–H st., cyclic ether), 1126 (C–O–C st., aryl ether), 3321 (O–H st., polyhydroxy), 1244 (C–O–C st., –OCH_3_); ^**1**^ **H-NMR (DMSO-d**_**6**_ **, δ ppm):** 7.45–7.49 7H, Ar–H), 7.49 (d, *J* = 8 Hz, 2H, Ar–H), 8.25 (s, 1H, NH of 2° amide), 4.20–4.22 (m, 5H, CH of tetrahydropyran), 2.10 {s, 4H, (OH)_4_}, 3.86 {s, 9H, (OCH_3_)_3_}; ^**13**^ **C-NMR (DMSO-d**_**6**_ **, δ ppm):** 21.3, 72.3, 76.4, 99.5, 56.2, 60.1, 104.2, 120.3, 125.4, 128.6, 128.9, 128.0, 130.1, 137.2, 152.3, 163.2**; MS ES** + (ToF): *m/z* 596 [M^+^+1].

**Compound 3** ***(7*** **-** ***Methyl*** **-** ***5*** **-** ***(4*** **-** ***nitrophenyl)*** **-** ***3*** **-** ***oxo*** **-** ***N*** **-** ***phenyl*** **-** ***2*** **-** ***((3,4,5,6*** **-** ***tetrahydroxytetrahydro*** **-** ***2H*** **-** ***pyran*** **-** ***2*** **-** ***yl)methylene)*** **-** ***1,2,3,5*** **-** ***tetrahydroimidazo [1,2*** **-** ***a]pyrimidine*** **-** ***6*** **-** ***carboxamide)*** **IR:** {3073 (C–H st.), 1598 (C=C st.), 716 (C–C st.) of aromatic ring}, 1630 (C=O st., 2˚amide), 1711 (C=O st., aryl ketone), 3354 (N–H st., 2° amide), {1711 (N=CH st.), 1347 (C–N st.) of pn}, 2779 (C–H st., cyclic ether), 1107 (C–O–C st., aryl ether), 3354 (O–H st., polyhydroxy), 1347 (NO_2_ st., phenyl nucleus), 854 (C–N st., C_6_H_5_NO_2_); ^**1**^ **H-NMR (DMSO-d**_**6**_ **, δ ppm):** 7.28–8.09 (m, 9H, Ar–H), 1.97 (s, 1H, NH), 8.10 (s, 1H, NH of 2° amide), 3.47–4.25 (m, 5H, CH of tetrahydropyran), 2.12 {s, 4H, (OH)_4_};^** 13**^ **C-NMR (DMSO-d**_**6**_ **, δ ppm):** 21.2, 71.3, 76.2, 98.5, 59.2, 120.3, 125.4, 128.7, 128.9, 128.0, 130.1, 137.2, 149.2, 152.3, 163.1; **MS ES** + (ToF): *m/z* 551 [M^+^+1].

**Compound 4** ***(5*** **-** ***(4*** **-** ***(Diethylamino)phenyl)*** **-** ***7*** **-** ***methyl*** **-** ***3*** **-** ***oxo*** **-** ***N*** **-** ***phenyl*** **-** ***2*** **-** ***((3,4,5,6*** **-** ***tetrahydroxytetrahydro*** **-** ***2H*** **-** ***pyran*** **-** ***2*** **-** ***yl)methylene)*** **-** ***1,2,3,5*** **-** ***tetrahydroimidazo[1,2*** **-** ***a]pyrimidine*** **-** ***6*** **-** ***carboxamide)*** **IR:** {2977 (C–H st.), 1590 (C=C st.), 708 (C–C st.) of aromatic ring}, 1650 (C=O st.,), 3283 (N–H st., 2° amide), {1650 (N=CH st., pn), 1255 (C–N st.) of pn}, 2738 (C–H st., cyclic ether), 1076 (C–O–C st., aryl ether), 3283 (O–H st., polyhydroxy), 2823 (C–H st., aliphatic chain), 1183 (C–C st., aliphatic chain); ^**1**^ **H-NMR (DMSO-d**_**6**_ **, δ ppm):** 6.63–7.49 (m, 9H, Ar–H), 2.11 (s, 1H, NH), 8.09 (s, 1H, NH of 2° amide), 6.7 (s, 1H, ethylene), 3.45–5.39 (m, 5H, CH of tetrahydropyran), 2.19 {s, 4H, (OH)_4_}, 1.19 {(t, 6H, (CH_3_)_2_, 3.43 (q, 4H, (CH_2_)_2_ of (C_2_H_5_)_2_}; ^**13**^ **C-NMR (DMSO-d**_**6**_ **, δ ppm):** 12.3, 21.3, 47.9, 72.3, 77.2, 98.5, 59.2, 112.7, 120.3, 121.9, 125.4, 128.5, 128.0, 128.4, 130.1, 137.2, 147.2, 152.3, 163.1; **MS ES** + (ToF): *m/z* 577 [M^+^+1].

**Compound 5** ***(7*** **-** ***Methyl*** **-** ***5*** **-** ***(3*** **-** ***nitrophenyl)*** **-** ***3*** **-** ***oxo*** **-** ***N*** **-** ***phenyl*** **-** ***2*** **-** ***((3,4,5,6*** **-** ***tetrahydroxytetrahydro*** **-** ***2H*** **-** ***pyran*** **-** ***2*** **-** ***yl)methylene)*** **-** ***1,2,3,5*** **-** ***tetrahydroimidazo[1,2*** **-** ***a]pyrimidine*** **-** ***6*** **-** ***carboxamide)*** **IR:** {3062 (C–H st.), 1597 (C=C st.), 693 (C–C st.) of aromatic ring}, 1630 (C=O st.,), 3307 (N–H st., 2° amide), {1630 (N=CH st.), 1330 (C–N st.) of pn}, 2779 (C–H st., cyclic ether), 1125 (C–O–C st., aryl ether), 3307 (O–H st., polyhydroxy), 1350 (NO_2_ st., phenyl nucleus), 841 (C–N st., C_6_H_5_NO_2_); ^**1**^ **H-NMR (DMSO-d**_**6**_ **, δ ppm):** 7.28–8.09 (m, 9H, Ar–H), 2.12 (s, 1H, NH), 8.10 (s, 1H, NH of 2^o^ amide), 3.47–4.23 (m, 5H, CH of tetrahydropyran), 2.19 {s, 4H, (OH)_4_}; ^**13**^ **C-NMR (DMSO-d**_**6**_ **, δ ppm):** 21.2, 72.3, 76.2, 98.5, 59.2, 120.3, 121.1, 125.4, 128.7, 128.9, 128.0, 129.1, 130.1, 133.3, 137.2, 144.2, 147.2, 152.3, 163.1; **MS ES** + (ToF): *m/z* 551 [M^+^+1].

**Compound 6** ***(7*** **-** ***Methyl*** **-** ***5*** **-** ***(2*** **-** ***nitrophenyl)*** **-** ***3*** **-** ***oxo*** **-** ***N*** **-** ***phenyl*** **-** ***2*** **-** ***((3,4,5,6*** **-** ***tetrahydroxytetrahydro*** **-** ***2H*** **-** ***pyran*** **-** ***2*** **-** ***yl)methylene)*** **-** ***1,2,3,5*** **-** ***tetrahydroimidazo[1,2*** **-** ***a]pyrimidine*** **-** ***6*** **-** ***carboxamide)*** **IR:** {2933 (C–H st.), 1597 (C=C st.), 691 (C–C st.) of aromatic ring}, 1630 (C=O st.,), 3385 (N–H st., 2° amide), {1630 (N=CH st., pn), 1245 (C–N st.) of pn}, 2779 (C–H st., cyclic ether), 1096 (C–O–C st., aryl ether), 3385 (O–H st., polyhydroxy), 1352 (NO_2_ st.), 855 (C–N st., C_6_H_5_NO_2_); ^**1**^ **H-NMR (DMSO-d**_**6**_ **, δ ppm):** 7.28–7.61 (m, 9H, Ar–H), 2.08(s, 1H, NH), 8.11 (s, 1H, NH of 2° amide), 1.88(s, 3H, CH_3_), 3.47–4.57 (m, 5H, CH of tetrahydropyran), 2.11 {s, 4H, (OH)_4_}; ^**13**^ **C-NMR (DMSO-d**_**6**_ **, δ ppm):** 21.4, 73.1, 76.2, 94.5, 120.5, 121.2, 125.4, 129.7, 127.2, 129.1, 127.1, 130.1, 131.3, 137.2, 146.2, 152.3, 162.1; **MS ES** + (ToF): *m/z* 551 [M^+^+1].

**Compound 7** ***(5*** **-** ***(4*** **-** ***(Dimethylamino)phenyl)*** **-** ***7*** **-** ***methyl*** **-** ***3*** **-** ***oxo*** **-** ***N*** **-** ***phenyl*** **-** ***2*** **-** ***((3,4,5,6*** **-** ***tetrahydroxytetrahydro*** **-** ***2H*** **-** ***pyran*** **-** ***2*** **-** ***yl)methylene)*** **-** ***1,2,3,5*** **-** ***tetrahydroimidazo[1,2*** **-** ***a]pyrimidine*** **-** ***6*** **-** ***carboxamide)*** **IR:** {3026 (C–H st.), 1559 (C=C st.), 714 (C–C st.) of aromatic ring}, 1595 (C=O st., 2° amide), 1711 (C=O st., aryl ketone), 3062 (N–H st., 2° amide), {1711 (N=CH st.), 1248 (C–N st.) of pn}, 2814 (C–H st., cyclic ether), 1070 (C–O–C st., aryl ether), 3399 (O–H st., polyhydroxy), 2934 (C–H st., aliphatic chain); ^**1**^ **H-NMR (DMSO-d**_**6**_ **, δ ppm):** 6.65–7.62 (m, 9H, Ar–H), 2.11 (s, 1H, NH), 8.09 (s, 1H, NH of 2° amide), 6.74 (s, 1H, ethylene), 3.47–4.41 (m, 5H, CH of tetrahydropyran), 2.19 {s, 4H, (OH)_4_}, 3.06 {s, 6H, of (CH_3_)_2_};^** 13**^ **C-NMR (DMSO-d**_**6**_ **, δ ppm):** 21.4, 41.0, 55.1, 70.1, 73.1, 76.2, 94.8, 120.5, 121.3, 124.1, 125.4, 129.0, 127.8, 127.1, 130.4, 132.6, 135.2, 147.2, 163.1; **MS ES** + (ToF): *m/z* 549 [M^+^+1].

**Compound 8** ***(5*** **-** ***(4*** **-** ***Hydroxyphenyl)*** **-** ***7*** **-** ***methyl*** **-** ***3*** **-** ***oxo*** **-** ***N*** **-** ***phenyl*** **-** ***2*** **-** ***((3,4,5,6*** **-** ***tetrahydroxytetrahydro*** **-** ***2H*** **-** ***pyran*** **-** ***2*** **-** ***yl)methylene)*** **-** ***1,2,3,5*** **-** ***tetrahydroimidazo[1,2*** **-** ***a]pyrimidine*** **-** ***6*** **-** ***carboxamide)*** **IR:** {3064 (C–H st.), 1596 (C=C st.), 714 (C–C st.) of aromatic ring}, 1596 (C=O st., 2° amide), 1712 (C=O st., aryl ketone), 3385 (N–H st., 2° amide), {1712 (N=CH st.), 1249 (C–N st.) of pn}, 2779 (C–H st., cyclic ether), 1083 (C–O–C st., aryl ether), 3385 (O–H st., polyhydroxy), 3385 (OH st., phenyl nucleus); ^**1**^ **H-NMR (DMSO-d**_**6**_ **, δ ppm):** 7.44–7.58 (m, 9H, Ar–H), 2.06 (s, 1H, NH), 8.07 (s, 1H, NH 2° amide), 3.45–4.96 (m, 5H, CH, tetrahydropyran), 2.16 {s, 4H, (OH)_4_}, 4.96 (s, 1H, Ar–OH); ^**13**^ **C-NMR (DMSO-d**_**6**_ **, δ ppm):** 21.2, 55.1, 71.1, 73.1, 76.2, 94.3, 113.6, 120.5, 121.3, 124.1, 125.4, 128.1, 129.0, 135.2, 147.2, 152.1, 156.2, 163.1; **MS ES** + (ToF): *m/z* 522 [M^+^+1].

**Compound 9** ***(5*** **-** ***(4*** **-** ***Hydroxy*** **-** ***3*** **-** ***methoxyphenyl)*** **-** ***7*** **-** ***methyl*** **-** ***3*** **-** ***oxo*** **-** ***N*** **-** ***phenyl*** **-** ***2*** **-** ***((3,4,5,6*** **-** ***tetrahydroxytetrahydro*** **-** ***2H*** **-** ***pyran*** **-** ***2*** **-** ***yl)methylene)*** **-** ***1,2,3,5*** **-** ***tetrahydroimidazo[1,2*** **-** ***a]pyrimidine*** **-** ***6*** **-** ***carboxamide)*** **IR:** {2967 (C–H st.), 1595 (C=C st.), 713 (C–C st.) of aromatic ring}, 1595 (C=O st.,), 3422 (N–H st., 2° amide), {1595 (N=CH st.), 1249 (C–N st.) of pn}, 2832 (C–H st., cyclic ether), 1070 (C–O–C st., aryl ether), 3422 (O–H st., polyhydroxy), 3422 (OH st., phenyl nucleus), 1249 (C–O–C st., –OCH_3_); ^**1**^ **H-NMR (DMSO-d**_**6**_ **, δ ppm):** 2.10 (s, 1H, NH), 5.71 (s, 1H, CH of pyrimidine), 3.46–4.85 (m, 5H, CH of tetrahydropyran), 2.18 {s, 4H, (OH)_4_}, 3.76 (s, 3H, OCH_3_); ^**13**^ **C-NMR (DMSO-d**_**6**_ **, δ ppm):** 21.3, 55.3, 56.1, 70.1, 73.1, 76.2, 94.8, 113.6, 120.6, 116.3, 121.4, 124.4, 125.1, 129.0, 130.2, 135.9, 136.2, 143.2, 151.2, 152.7, 162.1; **MS ES** + (ToF): *m/z* 552 [M^+^+1].

**Compound 10** ***(5*** **-** ***(2,4*** **-** ***Dichlorophenyl)*** **-** ***7*** **-** ***methyl*** **-** ***3*** **-** ***oxo*** **-** ***N*** **-** ***phenyl*** **-** ***2*** **-** ***((3,4,5,6*** **-** ***tetrahydroxytetrahydro*** **-** ***2H*** **-** ***pyran*** **-** ***2*** **-** ***yl)methylene)*** **-** ***1,2,3,5*** **-** ***tetrahydroimidazo[1,2*** **-** ***a]pyrimidine*** **-** ***6*** **-** ***carboxamide)*** **IR:** {2834 (C–H st.), 1594 (C=C st.), 703 (C–C st.) of aromatic ring}, 1594 (C=O st.,), 3380 (N–H st., 2° amide), {1594 (N=CH st.), 1350 (C–N st.) of pn}, 2735 (C–H st., cyclic ether), 1090 (C–O–C st., aryl ether), 3380 (O–H st., polyhydroxy), 758 (C–Cl st., phenyl nucleus); ^**1**^ **H-NMR (DMSO-d**_**6**_ **, δ ppm):** 6.94–7.50 (m, 8H, Ar–H), 2.08 (s, 1H, NH), 8.10 (s, 1H, NH of 2° amide), 6.21 (s, 1H, ethylene), 3.47–5.00 (m, 5H, CH of tetrahydropyran), 2.12 {s, 4H, (OH)_4_};^** 13**^ **C-NMR (DMSO-d**_**6**_ **, δ ppm):** 21.2, 45.2, 70.0, 73.1, 76.2, 94.9, 120.6, 121.4, 124.4, 125.1, 126.2, 129.0, 130.2, 133.4, 135.2, 140.2, 146.2, 152.7, 162.1, 163.3; **MS ES** + (ToF): *m/z* 574 [M^+^+1].

**Compound 11** ***(5*** **-** ***(2*** **-** ***Methoxyphenyl)*** **-** ***7*** **-** ***methyl*** **-** ***3*** **-** ***oxo*** **-** ***N*** **-** ***phenyl*** **-** ***2*** **-** ***((3,4,5,6*** **-** ***tetrahydroxytetrahydro*** **-** ***2H*** **-** ***pyran*** **-** ***2*** **-** ***yl)methylene)*** **-** ***1,2,3,5*** **-** ***tetrahydroimidazo[1,2*** **-** ***a]pyrimidine*** **-** ***6*** **-** ***carboxamide)*** **IR:** {3063 (C–H st.), 1595 (C=C st.), 710 (C–C st.) of aromatic ring}, 1630 (C=O st.,), 3397 (N–H st., 2° amide), {1630 (N=CH st.), 1247 (C–N st.) of pn}, 2832 (C–H st., cyclic ether), 1050 (C–O–C st., aryl ether), 3397 (O–H st., polyhydroxy), 1247 (C–O–C st., –OCH_3_); ^**1**^ **H-NMR (DMSO-d**_**6**_ **, δ ppm):** 6.89–7.58 (m, 9H, Ar–H), 2.04 (s, 1H, NH), 6.88 (s, 1H, ethylene), 3.44–4.97 (m, 5H, CH of tetrahydropyran), 2.07 {s, 4H, (OH)_4_}, 3.70 (s, 3H, OCH_3_); ^**13**^ **C-NMR (DMSO-d**_**6**_ **, δ ppm):** 21.1, 45.2, 70.3, 73.1, 76.2, 94.8, 114.1, 120.6, 121.4, 124.4, 125.1, 127.2, 128.2, 129.0, 130.2, 135.2, 140.2, 146.2, 156.7, 162.1, 163.3; **MS ES** + (ToF): *m/z* 536 [M^+^+1].

**Compound 12** ***(5*** **-** ***(3*** **-** ***Bromophenyl)*** **-** ***7*** **-** ***methyl*** **-** ***3*** **-** ***oxo*** **-** ***N*** **-** ***phenyl*** **-** ***2*** **-** ***((3,4,5,6*** **-** ***tetrahydroxytetrahydro*** **-** ***2H*** **-** ***pyran*** **-** ***2*** **-** ***yl)methylene)*** **-** ***1,2,3,5*** **-** ***tetrahydroimidazo[1,2*** **-** ***a]pyrimidine*** **-** ***6*** **-** ***carboxamide)*** **IR:** {3064 (C–H st.), 1596 (C=C st.), 712 (C–C st.) of aromatic ring}, 1596 (C=O st.,), 3386 (N–H st., 2° amide), {1596 (N=CH st.), 1253 (C–N st.) of pn}, 2832 (C–H st., cyclic ether), 1071 (C–O–C st., aryl ether), 3386 (O–H st., polyhydroxy), 510 (C–Br st.); ^**1**^ **H-NMR (DMSO-d**_**6**_ **, δ ppm):** 7.43–7.63 (m, 9H, Ar–H), 7.63 (d, *J* = 8 Hz, 2H, Ar–H), 8.09 (s, 1H, NH of 2° amide), 1.97 (s, 1H, NH), 1.84 (s, 3H, CH_3_), 6.18 (s, 1H, CH of ethylene), 3.47–4.38 (m, 5H, CH of tetrahydropyran), 2.11 {s, 4H, (OH)_4_}; ^**13**^ **C-NMR (DMSO-d**_**6**_ **, δ ppm):** 21.4, 54.8, 70.3, 73.1, 76.1, 94.7, 120.6, 121.4, 124.4, 125.1, 126.2, 129.0, 130.2, 135.2, 145.2, 146.2, 152.3, 162.1, 163.3; **MS ES** + (ToF): *m/z* 584 [M^+^+1].

**Compound 13** ***(5*** **-** ***(3*** **-** ***Methoxyphenyl)*** **-** ***7*** **-** ***methyl*** **-** ***3*** **-** ***oxo*** **-** ***N*** **-** ***phenyl*** **-** ***2*** **-** ***((3,4,5,6*** **-** ***tetrahydroxytetrahydro*** **-** ***2H*** **-** ***pyran*** **-** ***2*** **-** ***yl)methylene)*** **-** ***1,2,3,5*** **-** ***tetrahydroimidazo[1,2*** **-** ***a]pyrimidine*** **-** ***6*** **-** ***carboxamide)*** **IR:** {3062 (C–H st.), 1595 (C=C st.), 712 (C–C st.) of aromatic ring}, 1631 (C=O st., 2° amide), 1716 (C=O st., aryl ketone), 3385 (N–H st., 2° amide), {1631 (N=CH st.), 1247 (C–N st.) of pn}, 2831 (C–H st., cyclic ether), 1070 (C–O–C st., aryl ether), 3385 (O–H st., polyhydroxy), 1247 (C–O–C st., –OCH_3_); ^**1**^ **H-NMR (DMSO-d**_**6**_ **, δ ppm):** 7.25–7.48 (m, 9H, Ar–H), 1.96 (s, 1H, NH), 8.0 (s, 1H, NH of 2° amide), 3.45–4.99 (m, 5H, CH of tetrahydropyran), 2.11 {s, 4H, (OH)_4_}, 3.76 (s, 3H, OCH_3_); ^**13**^ **C-NMR (DMSO-d**_**6**_ **, δ ppm):** 21.3, 55.2, 55.8, 70.3, 73.1, 76.2, 94.9, 111.0, 197.0, 120.6, 121.5, 124.4, 125.1, 129.0, 130.2, 133.4, 135.2, 140.2, 146.2, 152.7, 162.1, 163.3; **MS ES** + (ToF): *m/z* 536 [M^+^+1].

**Compound 14** ***(5*** **-** ***(4*** **-** ***Bromophenyl)*** **-** ***7*** **-** ***methyl*** **-** ***3*** **-** ***oxo*** **-** ***N*** **-** ***phenyl*** **-** ***2*** **-** ***((3,4,5,6*** **-** ***tetrahydroxytetrahydro*** **-** ***2H*** **-** ***pyran*** **-** ***2*** **-** ***yl)methylene)*** **-** ***1,2,3,5*** **-** ***tetrahydroimidazo[1,2*** **-** ***a]pyrimidine*** **-** ***6*** **-** ***carboxamide)*** **IR:** {3058 (C–H st.), 1595 (C=C st.), 709 (C–C st.) of aromatic ring}, 1631 (C=O st., 2° amide), 1715 (C=O st., aryl ketone), 3333 (N–H st., 2° amide), {1631 (N=CH st.), 1315 (C–N st.) of pn}, 2831 (C–H st., cyclic ether), 1072 (C–O–C st., aryl ether), 3333 (O–H st., polyhydroxy), 509 (C–Br st.); ^**1**^ **H-NMR (DMSO-d**_**6**_ **, δ ppm):** 7.14–7.64 (m, 9H, Ar–H), 7.66 (d, *J* = 8 Hz, 2H, Ar–H), 8.1 (s, 1H, NH of 2° amide), 2.19 (s, 1H, NH), 1.82 (s, 3H, CH_3_), 3.47–5.00 (m, 5H, CH of tetrahydropyran), 2.13 {s, 4H, (OH)_4_}; ^**13**^ **C-NMR (DMSO-d**_**6**_ **, δ ppm):** 21.3, 55.0, 70.3, 73.1, 76.2, 94.8, 197.0, 120.6, 121.5, 124.4, 125.1, 129.0, 130.2, 131.1, 133.4, 135.7, 142.2, 146.2, 152.7, 162.1, 163.3; **MS ES** + (ToF): *m/z* 584 [M^+^+1].

**Compound 15 (** ***5*** **-** ***(5*** **-** ***Bromo*** **-** ***2*** **-** ***hydroxyphenyl)*** **-** ***7*** **-** ***methyl*** **-** ***3*** **-** ***oxo*** **-** ***N*** **-** ***phenyl*** **-** ***2*** **-** ***((3,4,5,6*** **-** ***tetrahydroxytetrahydro*** **-** ***2H*** **-** ***pyran*** **-** ***2*** **-** ***yl)methylene)*** **-** ***1,2,3,5*** **-** ***tetrahydroimidazo[1,2*** **-** ***a]pyrimidine*** **-** ***6*** **-** ***carboxamide)*** **IR:** {3062 (C–H st.), 1596 (C=C st.), 691 (C–C st.) of aromatic ring}, 1631 (C=O st., 2° amide), 1712 (C=O st., aryl ketone), 3332 (N–H st., 2° amide), {1631 (N=CH st.), 1282 (C–N st.) of pn}, 2832 (C–H st., cyclic ether), 1070 (C–O–C st., aryl ether), 3332 (O–H st., polyhydroxy), 3332 (OH st., phenyl), 543 (C–Br st.); ^**1**^ **H-NMR (DMSO-d**_**6**_ **, δppm):** 7.29–7.63 (m, 8H, Ar–H), 7.49 (d, *J* = 8 Hz, 2H, Ar–H), 2.13 (s, 1H, NH), 8.1(s, 1H, NH of 2° amide), 1.71 (s, 3H, CH_3_), 6.6 (s, 1H of ethylene), 3.79–5.12 (m, 5H, CH of tetrahydropyran), 1.98 {s, 4H, (OH)_4_}, 5.07 (s, 1H, of Ar–OH); ^**13**^ **C-NMR (DMSO-d**_**6**_ **, δ ppm):** 21.3, 44.2, 70.3, 73.4, 76.2, 94.9, 117.0, 115.3, 120.6, 121.4, 124.1, 125.1, 129.0, 130.2, 131.2, 133.4, 135.2, 146.2, 153.2, 162.1, 163.3; **MS ES** + (ToF): *m/z* 601 [M^+^+1].

**Compound 16** ***(5*** **-** ***(4*** **-** ***Methoxyphenyl)*** **-** ***7*** **-** ***methyl*** **-** ***3*** **-** ***oxo*** **-** ***N*** **-** ***phenyl*** **-** ***2*** **-** ***((3,4,5,6*** **-** ***tetrahydroxytetrahydro*** **-** ***2H*** **-** ***pyran*** **-** ***2*** **-** ***yl)methylene)*** **-** ***1,2,3,5*** **-** ***tetrahydroimidazo[1,2*** **-** ***a]pyrimidine*** **-** ***6*** **-** ***carboxamide)*** **IR:** {3062 (C–H st.), 1595 (C=C st.), 691 (C–C st.) of aromatic ring}, 1630 (C=O st.,), 3385 (N–H st., 2° amide), {1630 (N=CH st.), 1247 (C–N st.) of pn}, 2831 (C–H st., cyclic ether), 1072 (C–O–C st., aryl ether), 3385 (O–H st., polyhydroxy), 1247 (C–O–C st., –OCH_3_); ^**1**^ **H-NMR (DMSO-d**_**6**_ **, δ ppm):** 7.28–7.45 (m, 9H, Ar–H), 8.04 (s, 1H, NH of 2^o^ amide), 4.15–4.21(m, 5H, CH of tetrahydropyran), 2.40 {s, 4H, (OH)_4_}, 3.44 (s, 3H, OCH_3_), 1.71 (s, 3H, CH_3_); ^**13**^ **C-NMR (DMSO-d**_**6**_ **, δ ppm):** 21.1, 55.0, 55.8, 70.3, 73.1, 76.2, 94.5, 114.0, 120.6, 121.5, 124.4, 125.1, 128.3, 129.0, 130.2, 135.2, 146.2, 152.7, 158.1, 162.1, 163.3; **MS ES** + (ToF): *m/z* 536 [M^+^+1].

**Compound 17** ***(5*** **-** ***(4*** **-** ***Formylphenyl)*** **-** ***7*** **-** ***methyl*** **-** ***3*** **-** ***oxo*** **-** ***N*** **-** ***phenyl*** **-** ***2*** **-** ***((3,4,5,6*** **-** ***tetrahydroxytetrahydro*** **-** ***2H*** **-** ***pyran*** **-** ***2*** **-** ***yl)methylene)*** **-** ***1,2,3,5*** **-** ***tetrahydroimidazo[1,2*** **-** ***a]pyrimidine*** **-** ***6*** **-** ***carboxamide)*** **IR:** {3063 (C–H st.), 1595 (C=C st.), 690 (C–C st.) of aromatic ring}, 1630 (C=O st.,), 3384 (N–H st., 2° amide), {1630 (N=CH st.), 1244 (C–N st.) of pn}, 2831 (C–H st., cyclic ether), 1071 (C–O–C st., aryl ether), 3384 (O–H st., polyhydroxy), 2716 (C–H st., CHO), 1364 (C–C st., CHO group); ^**1**^ **H-NMR (DMSO-d**_**6**_ **, δ ppm):** 7.23–7.62 (m, 9H, Ar–H), 1.97 (s, 1H, NH), 8.16 (s, 1H, NH of 2° amide), 3.47–4.99 (m, 5H, CH of tetrahydropyran), 2.12 {s, 4H, (OH)_4_};^** 13**^ **C-NMR (DMSO-d**_**6**_ **, δ ppm):** 21.3, 55.3, 70.4, 73.1, 76.2, 94.8, 120.6, 121.4, 124.4, 125.1, 127.3, 129.0, 130.2, 134.3, 135.2, 146.2, 149.3, 152.7, 162.1, 163.3, 192.2; **MS ES** + (ToF): *m/z* 534 [M^+^+1].

**Compound 18** ***(5*** **-** ***(3*** **-** ***Chlorophenyl)*** **-** ***7*** **-** ***methyl*** **-** ***3*** **-** ***oxo*** **-** ***N*** **-** ***phenyl*** **-** ***2*** **-** ***((3,4,5,6*** **-** ***tetrahydroxytetrahydro*** **-** ***2H*** **-** ***pyran*** **-** ***2*** **-** ***yl)methylene)*** **-** ***1,2,3,5*** **-** ***tetrahydroimidazo[1,2*** **-** ***a]pyrimidine*** **-** ***6*** **-** ***carboxamide)*** **IR:** {3057 (C–H st.), 1596 (C=C st.), 689 (C–C st.) of aromatic ring}, 1666 (C=O st., 2° amide), 1717 (C=O st., aryl ketone), 3327 (N–H st., 2° amide), {1666 (N=CH st.), 1315 (C–N st.) of pn}, 2830 (C–H st., cyclic ether), 1082 (C–O–C st., aryl ether), 3327 (O–H st., polyhydroxy), 758 (C–Cl st.); ^**1**^ **H-NMR (DMSO-d**_**6**_ **, δ ppm):** 7.28–7.63(m, 9H, Ar–H), 7.52 (d, *J* = 4 Hz, 2H, Ar–H), 2.13 (s, 1H, NH), 8.11(s, 1H, NH of 2° amide), 1.85 (s, 3H, CH_3_), 3.47–5.00 (m, 5H, CH of tetrahydropyran), 1.97 {s, 4H, (OH)_4_}; ^**13**^ **C-NMR (DMSO-d**_**6**_ **, δ ppm):** 21.3, 55.3, 70.4, 73.1, 76.2, 94.8, 120.6, 121.4, 124.4, 125.1, 126.2, 129.0, 130.2, 135.2, 144.2, 146.3, 152.7, 162.1, 163.3; **MS ES** + (ToF): *m/z* 540 [M^+^+1].

**Compound 19** ***(7*** **-** ***Methyl*** **-** ***3*** **-** ***oxo*** **-** ***N,5*** **-** ***diphenyl*** **-** ***2*** **-** ***((3,4,5,6*** **-** ***tetrahydroxytetrahydro*** **-** ***2H*** **-** ***pyran*** **-** ***2*** **-** ***yl)methylene)*** **-** ***1,2,3,5*** **-** ***tetrahydroimidazo[1,2*** **-** ***a]pyrimidine*** **-** ***6*** **-** ***carboxamide):*** **IR-{**3057 (C–H st.), 1594 (C=C st.), 706 (C–C st.) of aromatic ring}, 1664 (C=O st., 2° amide), 1714 (C=O st., aryl ketone), 3335 (N–H st., 2° amide), {1664 (N=CH st.), 1315 (C–N st.) of pn}, 2831 (C–H st., cyclic ether), 1073 (C–O–C st., aryl ether), 3335 (O–H st., polyhydroxy), 3335 (OH st., phenyl), 2616 (C-H st., CHO), 1364 (C–C st., C_6_H_5_CHO); ^**1**^ **H-NMR (DMSO-d**_**6**_ **, δ ppm):** 7.48–7.64 (m, 10H, Ar–H), 1.96 (s, 1H, NH), 8.1 (s, 1H, NH of 2° amide), 1.84 (s, 3H, CH_3_), 3.75–4.24 (m, 5H, CH of tetrahydropyran), 2.12 {s, 4H, (OH)_4_}; ^**13**^ **C-NMR (DMSO-d**_**6**_ **, δ ppm):** 21.0, 55.1, 70.3, 73.1, 76.2, 94.2, 120.5, 121.2, 124.4, 125.1, 126.2, 128.3, 129.0, 130.4, 135.2, 143.2, 146.3, 152.7, 162.1, 163.3; **MS ES** + (ToF): *m/z* 506[M^+^+1].

**Compound 20** ***(5*** **-** ***(4*** **-** ***Chlorophenyl)*** **-** ***7*** **-** ***methyl*** **-** ***3*** **-** ***oxo*** **-** ***N*** **-** ***phenyl*** **-** ***2*** **-** ***((3,4,5,6*** **-** ***tetrahydroxytetrahydro*** **-** ***2H*** **-** ***pyran*** **-** ***2*** **-** ***yl)methylene)*** **-** ***1,2,3,5*** **-** ***tetrahydroimidazo[1,2*** **-** ***a]pyrimidine*** **-** ***6*** **-** ***carboxamide)*** **IR:** 2958 (C–H st.), 1594 (C=C st.), 709 (C–C st.) of aromatic ring}, 1630 (C=O st.,), 3420 (N–H st., 2° amide), {1630 (N=CH st.), 1177 (C–N st.) of pn}, 2831 (C–H st., cyclic ether), 1090 (C–O–C st., aryl ether), 3420 (O–H st., polyhydroxy), 775 (C–Cl st.); ^**1**^ **H-NMR (DMSO-d**_**6**_ **, δ ppm):** 7.29–7.64 (m, 9H, Ar–H), 2.07 (s, 1H, NH), 8.0(s, 1H, NH of 2° amide), 1.83 (s, 3H, CH_3_), 6.08 (s, 1H of ethylene), 3.47–4.87 (m, 5H of CH of tetrahydropyran), 2.09 {s, 4H, (OH)_4_};^** 13**^ **C-NMR (DMSO-d**_**6**_ **, δ ppm):** 21.3, 55.0, 70.4, 73.1, 77.2, 94.8, 120.6, 121.4, 124.4, 125.1, 128.3, 128.5, 129.0, 130.2, 135.2, 141.4, 146.2, 152.7, 162.1, 163.1; **MS ES** + (ToF): *m/z* 540 [M^+^+1].

**Compound 21** ***(5*** **-** ***(3*** **-** ***Ethoxy*** **-** ***4*** **-** ***hydroxyphenyl)*** **-** ***7*** **-** ***methyl*** **-** ***3*** **-** ***oxo*** **-** ***N*** **-** ***phenyl*** **-** ***2*** **-** ***((3,4,5,6*** **-** ***tetrahydroxytetrahydro*** **-** ***2H*** **-** ***pyran*** **-** ***2*** **-** ***yl)methylene)*** **-** ***1,2,3,5*** **-** ***tetrahydroimidazo[1,2*** **-** ***a]pyrimidine*** **-** ***6*** **-** ***carboxamide)*** **IR:** {3027 (C–H st.), 1559 (C=C st.), 710 (C–C st.) of aromatic ring}, 1594 (C=O st., 2° amide), 1713 (C=O st., aryl ketone), 3416 (N–H st., 2° amide), {1713 (N=CH st.), 1316 (C–N st.) of pn}, 2831 (C–H st., cyclic ether), 1071 (C–O–C st., aryl ether), 3416 (O–H st., polyhydroxy), 3416 (OH st., phenyl nucleus), 2831 (C–H st., aliphatic chain), 1175 (C–C st., aliphatic chain); ^**1**^ **H-NMR (DMSO-d**_**6**_ **, δ ppm):** 7.28–7.63 (m, 8H, Ar–H), 7.50 (d, *J* = 8 Hz, 2H, Ar–H), 2.12 (s, 1H, NH), 8.10 (s, 1H, NH 2° amide), 1.83 (s, 3H, CH_3_), 3.47–4.99 (m, 5H, CH of tetrahydropyran), 1.97 {s, 4H, (OH)_4_}, 1.33 (dt, J = 8 Hz, 3H, CH_3_), 4.21 (d, J = 8 Hz, 2H, CH_2_); ^**13**^ **C-NMR (DMSO-d**_**6**_ **, δ ppm):** 21.5, 65.1, 70.2, 73.4, 76.2, 77.5, 94.7, 116.3, 120.1, 121.5, 130.6, 162.3, 143.1, 146.4, 163.4, 121.3,124.4, 125.8, 135.4, 129.1, 128.8, 136.5, 137.7, 148.3; **MS ES** + (ToF): *m/z* 566 [M^+^+1].

**Compound 22** ***(5*** **-** ***(2*** **-** ***Hydroxyphenyl)*** **-** ***7*** **-** ***methyl*** **-** ***3*** **-** ***oxo*** **-** ***N*** **-** ***phenyl*** **-** ***2*** **-** ***((3,4,5,6*** **-** ***tetrahydroxytetrahydro*** **-** ***2H*** **-** ***pyran*** **-** ***2*** **-** ***yl)methylene)*** **-** ***1,2,3,5*** **-** ***tetrahydroimidazo[1,2*** **-** ***a]pyrimidine*** **-** ***6*** **-** ***carboxamide)*** **IR:** {3060 (C–H st.), 1596 (C=C st.), 691 (C–C st.) of aromatic ring}, 1630 (C=O st.,), 3333 (N–H st., 2° amide), {1630 (N=CH st.), 1294 (C–N st.) of pn}, 2832 (C–H st., cyclic ether), 1103 (C–O–C st., aryl ether), 3333 (O–H st., polyhydroxy), 3333 (OH st., phenyl nucleus); ^**1**^ **H-NMR (DMSO-d**_**6**_ **, δ ppm):** 7.28 (m, 9H, Ar–H), 8.13 (s, 1H, NH of 2° amide), 3.47–4.24 (m, 5H, CH of tetrahydropyran), 2.19 {s, 4H, (OH)_4_};^** 13**^ **C-NMR (DMSO-d**_**6**_ **, δ ppm):** 21.2, 44.2, 70.0, 73.1, 76.2, 94.9, 115.8, 120.6, 130.4, 121.4, 122.6, 124.4, 125.1, 128.7, 129.0, 130.3, 135.2, 146.2, 152.7, 154.2, 162.4; **MS ES** + (ToF): *m/z* 522 [M^+^+1].

**Compound 23** ***(5*** **-** ***(2*** **-** ***Chlorophenyl)*** **-** ***7*** **-** ***methyl*** **-** ***3*** **-** ***oxo*** **-** ***N*** **-** ***phenyl*** **-** ***2*** **-** ***((3,4,5,6*** **-** ***tetrahydroxytetrahydro*** **-** ***2H*** **-** ***pyran*** **-** ***2*** **-** ***yl)methylene)*** **-** ***1,2,3,5*** **-** ***tetrahydroimidazo[1,2*** **-** ***a]pyrimidine*** **-** ***6*** **-** ***carboxamide)*** **IR:** {3059 (C–H st.), 1594 (C=C st.), 708 (C–C st.) of aromatic ring}, 1594 (C=O st.,), 3383 (N–H st., 2° amide), {1594 (N=CH st.), 1316 (C–N st.) of pn}, 2830 (C–H st., cyclic ether), 1071 (C–O–C st., aryl ether), 3383 (O–H st., polyhydroxy), 758 (C–Cl st.); ^**1**^ **H-NMR (DMSO-d**_**6**_ **, δ ppm):** 7.35–7.64 (m, 9H, Ar–H), 2.13 (s, 1H, NH), 8.1 (s, 1H, NH of 2° amide), 3.47–4.69 (m, 5H, CH of tetrahydropyran), 2.13 {s, 4H, (OH)_4_}; ^**13**^ **C-NMR (DMSO-d**_**6**_ **, δ ppm):** 21.2, 45.9, 70.0, 73.1, 76.1, 94.8, 120.5, 130.4, 121.6, 122.6, 125.1, 126.1, 128.7, 129.0, 135.8, 146.2, 152.7, 162.6, 163.2; **MS ES** + (ToF): *m/z* 540 [M^+^+1].

**Compound 24** ***(7*** **-** ***Methyl*** **-** ***3*** **-** ***oxo*** **-** ***N*** **-** ***phenyl*** **-** ***5*** **-** ***((E)*** **-** ***styryl)*** **-** ***2*** **-** ***((3,4,5,6*** **-** ***tetrahydroxytetrahydro*** **-** ***2H*** **-** ***pyran*** **-** ***2*** **-** ***yl)methylene)*** **-** ***1,2,3,5*** **-** ***tetrahydroimidazo[1,2*** **-** ***a]pyrimidine*** **-** ***6*** **-** ***carboxamide)*** **IR:** {2967 (C–H st.), 1594 (C=C st.), 710 (C–C st.) of aromatic ring}, 1630 (C=O st.,), 3422 (N–H st., 2° amide), {1630 (N=CH st.), 1271 (C–N st.) of pn}, 2831 (C–H st., cyclic ether), 1070 (C–O–C st., aryl ether), 3422 (O–H st., polyhydroxy), {2831 (C–H st.), 1176 (C–C st.), 1630 (C=C st.) of aliphatic chain}; ^**1**^ **H-NMR (DMSO-d**_**6**_ **, δ ppm):** 7.33-7.63 (m, 10H, Ar–H), 2.09 (s, 1H, NH), 8.09 (s, 1H, NH of 2° amide), 6.69 (s, 1H, ethylene), 3.48–5.04 (m, 5H, CH of tetrahydropyran), 2.1 {s, 4H, (OH)_4_}, 6.16 {d, 1H,(CH)_2_; 6.51 (d, 1H, (CH)_b_ of aliphatic chain}; ^**13**^ **C-NMR (DMSO-d**_**6**_ **, δ ppm):** 21.5, 73.4, 77.5, 79.3, 98.7, 122.5, 130.6, 162.3, 146.4, 163.4, 121.3, 123.4, 125.8, 137.4, 127.1, 128.8, 133.6, 136.5, 137.7 153.6, 119.3; **MS ES** + (ToF): *m/z* 532 [M^+^+1].

## Conclusions

Summarizing, we may conclude that the synthesized compounds **12, 14, 15, 18** and **21** displayed appreciable antibacterial and antifungal activities and compounds **2** and **16** exhibited excellent in vitro antioxidant activity due to the presence of electron releasing groups on benzylidene portion and anticancer activity indicated that compound **23** was found to be most active against human breast (MCF-7) cancer cell line due to the presence of electron withdrawing groups (*o*-Cl) on benzylidene portion. These compounds may be used as lead for the development of novel therapeutic agents.
